# Metallothionein Cup1 attenuates nitrosative stress in the yeast *Saccharomyces cerevisiae*

**DOI:** 10.15698/mic2023.08.802

**Published:** 2023-07-10

**Authors:** Yuki Yoshikawa, Ryo Nasuno, Naoki Takaya, Hiroshi Takagi

**Affiliations:** 1 Division of Biological Science, Graduate School of Science and Technology, Nara Institute of Science and Technology, 8916-5 Takayama, Ikoma, Nara 630-0192, Japan.; 2 Present address: Department of Biotechnology, Faculty of Bioresource Science, Akita Prefectural University, 241-438 Kaidoubata-Nishi, Shimoshinjo-Nakano, Akita, Akita 010-0195, Japan.; 3 Present address: Engineering Biology Research Center, Kobe University, 7-1-48, Minatojima Minami-machi, Chuo-ku, Kobe, Hyogo, 650-0047, Japan.; 4 Faculty of Life and Environmental Sciences, Microbiology Research Center for Sustainability, University of Tsukuba, 1-1-1 Tennodai, Tsukuba, Ibaraki 305-8572, Japan.

**Keywords:** metallothionein, nitrosative stress, yeast, stress tolerance

## Abstract

Metallothionein (MT), which is a small metal-binding protein with cysteine-rich motifs, functions in the detoxification of heavy metals in a variety of organisms. Even though previous studies suggest that MT is involved in the tolerance mechanisms against nitrosative stress induced by toxic levels of nitric oxide (NO) in mammalian cells, the physiological functions of MT in relation to NO have not been fully understood. In this study, we analyzed the functions of MT in nitrosative stress tolerance in the yeast *Saccharomyces cerevisiae*. Our phenotypic analyses showed that deletion or overexpression of the MT-encoding gene, *CUP1*, led to higher sensitivity or tolerance to nitrosative stress in *S. cerevisiae* cells, respectively. We further examined whether the yeast MT Cup1 in the cell-free lysate scavenges NO. These results showed that the cell-free lysate containing a higher level of Cup1 degraded NO more efficiently. On the other hand, the transcription level of *CUP1* was not affected by nitrosative stress treatment. Our findings suggest that the yeast MT Cup1 contributes to nitrosative stress tolerance, possibly as a constitutive rather than an inducible defense mechanism.

## INTRODUCTION

Nitric oxide (NO), which is one of the reactive nitrogen species (RNS), functions as a ubiquitous signaling molecule in a variety of biological phenomena [1, 2]. The effects of NO on cells can be either beneficial or harmful depending on its concentration. At appropriate levels, NO exerts physiological functions in humans, plants, and microorganisms [3]. Thus, NO induces the relaxation of vascular smooth muscle cells [4], and NO is involved in high temperature stress tolerance in yeast [5, 6]. But in excess amounts, NO can induce cellular damage and/or cell death, which is called nitrosative stress. For example, NO induces genetic mutations through DNA strand breakage and/or purine deamination [7]. Due to its high reactivity, NO synthesized by macrophages functions as a weapon to kill pathogenic microbes during infection [8, 9]. Therefore, mechanisms to control the concentration of NO or attenuate nitrosative stress are important for the growth and survival of various organisms.

A wide variety of NO-degrading and detoxification mechanisms have been reported. NO is oxidized or reduced to NO_3_^-^ or N_2_O under aerobic or anaerobic conditions by flavohemoglobin (fHb), respectively [10, 11]. Catalase degrades NO to nitrite in the presence of H_2_O_2_ even though NO inhibits the activity of catalase *via* its competitive binding against H_2_O_2_ [12]. Recently, we found that catalase was important for nitrosative stress resistance in NADPH-depleted yeast cells [13]. In another recent study, we found that GTP cyclohydrolase II (GCH2), which is the first-step enzyme in the riboflavin biosynthesis pathway, contributes to the nitrosative stress tolerance in yeast through mechanisms in which the reaction product of GCH2, 2,5-diamino-6-(5-phospho-D-ribosylamino)-pyrimidin-4(3H)-one, scavenges RNS [14].

NO reacts with thiol-containing compounds and then forms the corresponding *S*-nitroso molecules. GSH, the reduced form of glutathione, is a thiol-containing peptide that is present in high concentrations in cells, and reacts preferentially with NO to form its *S*-nitroso derivative, *S*-nitrosoglutathione (GSNO). GSNO is reductively degraded to GSH and ammonia by GSNO reductase (GSNOR), thioredoxin (Trx), and thioredoxin reductase (Trr) using NADPH as an electron donor [15]. In the filamentous fungus *Aspergillus nidulans*, an NO-inducible oligopeptide containing many cysteine residues, nitrosothionein, whose transcription is upregulated by RNS, traps NO by its cysteine residues to attenuate nitrosative stress with the aid of Trx, Trr, and NADPH [16]. These results indicate that the intracellular thiol-containing compounds are extremely important for the nitrosative stress tolerance [15].

Metallothionein (MT), which has been found in microbes, plants, and mammals, is a small cysteine-rich protein harboring conserved amino acid sequence motifs such as CXC, CXXC, and CCXCC [17–19]. MT binds to heavy metals such as zinc, cadmium, and copper *via* its conserved cysteine-rich motifs to detoxify them [20, 21]. MT exists as a complex with transition metal ions like zinc and cadmium and then releases them through reaction with reactive oxygen species or RNS, respectively, which contributes to transition metal homeostasis [22–24]. The previous studies reported that overproduction of MT enhanced the RNS resistance of mice cells [25, 26]. NO also upregulates the expression level of the MT-encoding gene in rat cells [27]. However, it is still unclear whether the physiological expression level of MT is involved in nitrosative stress tolerance in mammalian cells.

In the yeast *Saccharomyces cerevisiae*, MTs are encoded by the *CUP1* or *CRS5* gene. *S. cerevisiae* harbors two copies of the *CUP1* gene, *CUP1-1* and *CUP1-2,* of which nucleotide sequences are almost completely identical from 1,533 bp upstream to 269 bp downstream of their coding regions. A previous report suggests that the *CUP1* genes are duplicated by segmental duplication in the eighth chromosome [28]. The expression of *CUP1* is induced by copper ion *via* activation of the copper responsive transcriptional activator Ace1 [29]. The Cup1 protein, the gene product of *CUP1*, functions in the detoxification of metal ions such as copper or cadmium by trapping them [30, 31]. *CUP1* is also induced by oxidative stress and contributes to oxidative stress tolerance [32]. On the other hand, it was reported that the expression of *CRS5* was repressed under oxidative condition [33]. Since NO functions as an oxidant and thus the response mechanisms against nirosative and oxidative stress partly overlap [13, 34], Cup1 is likely to be important for nitrosative stress tolerance, even though the physiological roles of Cup1 in yeast under nitrosative stress conditions have not been fully understood.

Here, we analyzed the growth phenotypes of *S. cerevisiae* cells deleting or overexpressing the *CUP1* gene under nitrosative stress conditions. In addition, we detected the NO scavenging activity in the Cup1-containing cell-free lysate. Our phenotypic and molecular analyses indicated that Cup1 contributes to the nitrosative stress tolerance by scavenging NO in *S. cerevisiae* cells.

## RESULTS

### Yeast metallothionein Cup1 contributes to nitrosative stress tolerance

**Figure 1:**
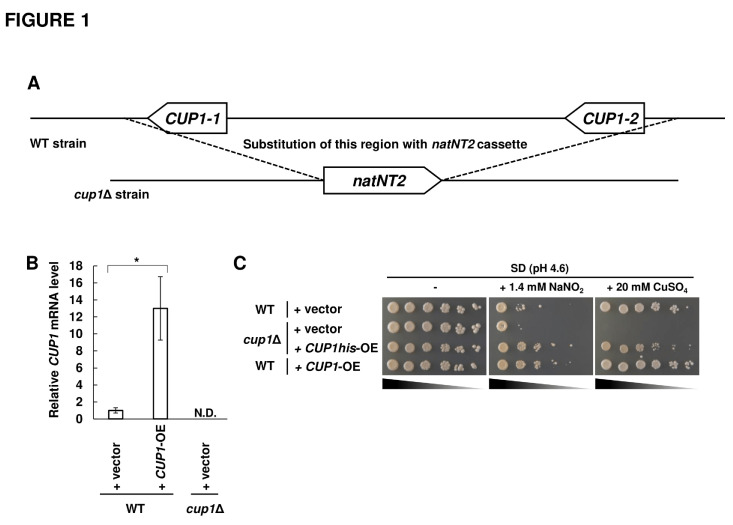
FIGURE 1: Growth phenotypes of *S. cerevisiae* cells. **(A)** Structure of the CUP1 locus on the genome of WT and *cup1*Δ strain. The DNA region from 1,533 bp upstream of *CUP1-1* coding region to 338 bp downstream of *CUP1-2* coding region was substituted with *natNT2* transformation cassette to construct the *cup1*Δ strain. **(B)** The *CUP1* mRNA was determined by RT-qPCR in each strain. The relative mRNA level was calculated with WT with an empty vector as 1.0 using *ACT1* gene as a reference. The values are the means and standard deviations from at least three independent experiments. Statistic significances of differences were analyzed by Student's *t*-test (*p < 0.05). **(C)** Spot assay of *S. cerevisiae* under stress conditions. Each strain was cultured until the exponential phase in SD liquid medium (pH 6.0) at 25°C, and the serial dilutions were spotted onto SD medium (pH 4.6) with or without of 1.4 mM NaNO_2,_ or 20 μM CuSO_4_ followed by incubation at 30°C for the indicated time. (**B, C**) The appropriate strains harboring an empty vector (+ vector), or overexpresses *CUP1* (+ *CUP1*-OE) or *CUP1his* (+ *CUP1his*-OE), were used.

In order to examine whether the yeast MT Cup1 protects cells from nitrosative stress, we analyzed the nitrosative stress resistance of yeast cells lacking or overexpressing *CUP1*. We constructed a strain lacking both *CUP1-1* and *CUP1-2* (*cup1*Δ strain) (**Figure 1A**) and a *CUP1-1*-overexpressing strain. The expression level of the *CUP1* gene in each strain was measured by quantitative RT-PCR (RT-qPCR) to confirm the deletion or overexpression of the *CUP1* gene (**Figure 1B**). As a result, the mRNA level of *CUP1* was undetectable in the *cup1*Δ strain, indicating that both the *CUP1-1* and *CUP1-2* genes were deleted. On the other hand, the *CUP1* transcription level was drastically increased in the *CUP1*-overexpressing strain compared with that in the wild-type (WT) strain, which demonstrates that *CUP1* was overexpressed as expected. Subsequently, the growth phenotypes of each strain were analyzed by spot assay (**Figure 1C**). Deletion or overexpression of the *CUP1* gene did not affect the growth of yeast strains on a minimal synthetic dextrose (SD) medium. When yeast strains were cultured with CuSO_4_, the *cup1*Δ strain did not grow at all, although the WT strain grew, indicating that *CUP1* is involved in the copper resistance, consistent with the previous report [30]. The compensation of copper sensitivity of *cup1*Δ strain by the overexpression of *CUP1his*, which encodes Cup1 fused with a hexa-histidine tag at its C-terminus (Cup1-His), demonstrated that Cup1-His normally functions in yeast cells. Next, yeast cells were grown on an acidified medium containing nitrite, under which condition nitrite is converted into RNS [35]. Importantly, the *cup1*Δ strain exhibited a growth defect compared with WT strain in the presence of RNS, which was recovered by the overexpression of *CUP1his*, indicating that *CUP1* under the control of its physiological expression system is required for nitrosative stress tolerance in yeast. Furthermore, the overexpression of *CUP1* improved the growth under nitrosative stress conditions compared with WT strain harboring an empty vector. These results indicated that the *CUP1* gene contributed to the nitrosative stress tolerance in a manner dependent on its expression level.

### Cup1 reduces the intracellular NO level by scavenging NO

**Figure 2:**
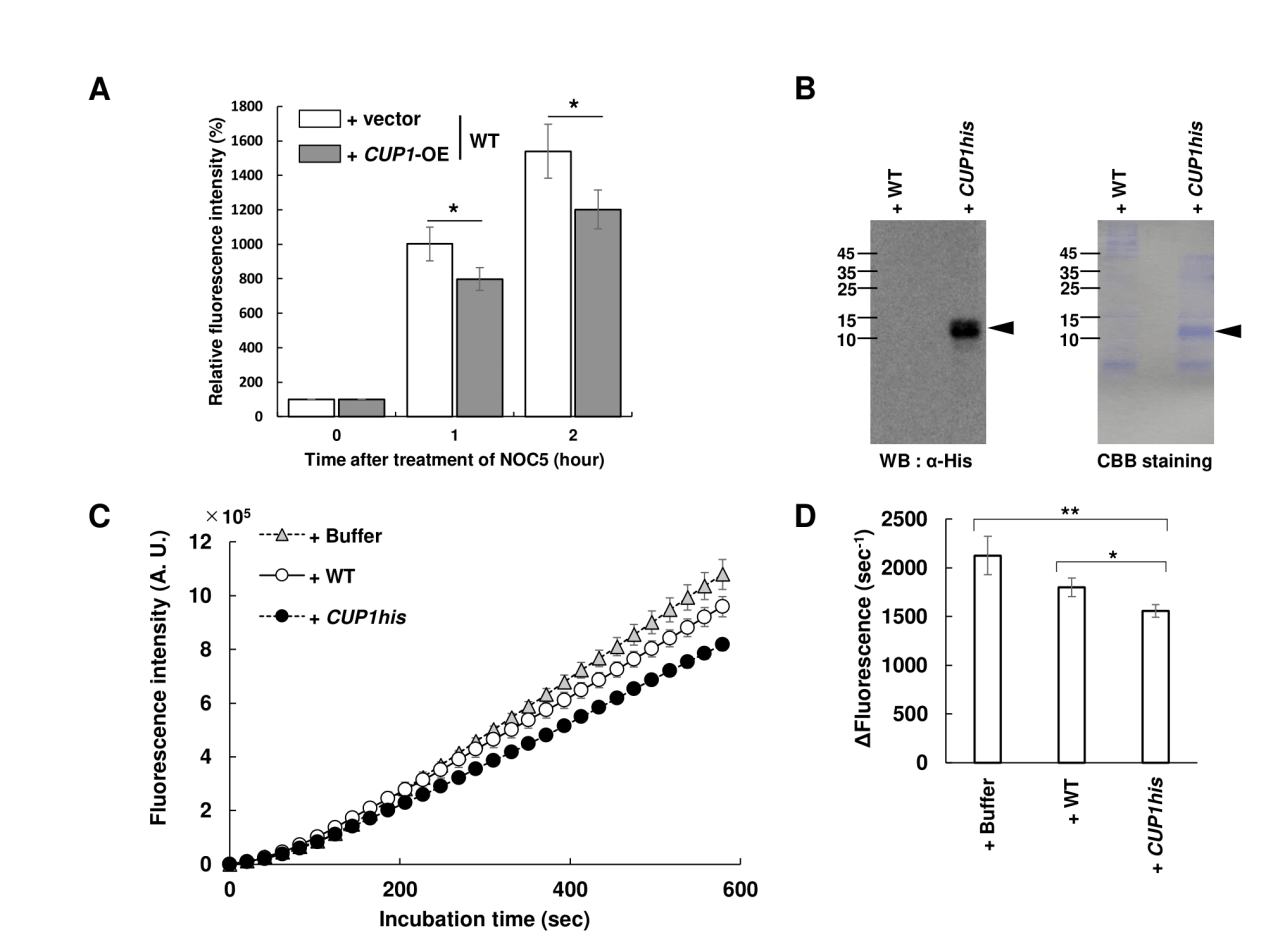
FIGURE 2: NO degradation by Cup1 in *S. cerevisiae* cells. **(A)** The intracellular NO level in *S. cerevisiae* cells was quantified. The WT strain harboring empty vector or overexpressing *CUP1* was cultured to the early exponential phase in SD medium (pH 6.0) at 25°C and then treated with DAF-FM DA. Subsequently, cells were exposed to NOC-5, followed by FCM. The relative fluorescence intensity was calculated with the fluorescence at 0 hour after the beginning of incubation as 100%. The means and standard deviations from at least three independent experiments were shown. Statistical significance of differences was analyzed by Student's *t* test (*p < 0.05, WT vs. *CUP1*-OE). (**B, C, D**) The yeast WT strain harboring a empty vector or the *cup1*Δ strain overexpressing *CUP1his* was shown as WT or *CUP1his*, respectively. **(B)** Detection and quantification of Cup1-His in the *CUP1his* strain. WT and *CUP1his* strain were cultured to the exponential phase in SD medium at 25°C, and the cell-free lysate was analyzed by SDS-PAGE followed by immunoblotting with anti-His antibody or CBB staining. An arrowhead in each picture indicates Cup1-His. **(C)** The inhibition of the time-dependent fluorescence increase mediated by Cup1-His. Each NOC-5 was reacted with DAF-FM in the absence or presence of the cell-free lysate from WT or *CUP1his* strain, and then the fluorescence intensity was monitored over time. The means and standard deviations from three independent experiments were shown. **(D)** The rate of fluorescence increases from 100 sec to 600 sec after reaction started were calculated. The means and standard deviations from three independent experiments were shown. Statistical significance in differences was analyzed by Student's *t* test (*p < 0.05, **p < 0.01).

We next analyzed the effect of Cup1 on the intracellular NO content in yeast cells. Yeast cells grown until the exponential growth phase were treated with a cell-permeable fluorescent NO probe, 4-amino-5-methylamino-2',7'-difluorofluorescein diacetate (DAF-FM DA), and then exposed to an NO donor 3-[2-hydroxy-1-(1-methylethyl)-2-nitrosohydrazino]-1-propanamine (NOC-5), followed by flow cytometry (**Figure 2A**). The results showed that the intracellular fluorescence intensity in WT cells increased over time, which was diminished by the overexpression of *CUP1*. This suggests that Cup1 decreases the intracellular NO level.

We then examined whether the Cup1 protein scavenges NO. For this analysis, we used the WT strain harboring an empty vector and the *cup1*Δ strain overexpressing *CUP1his*, which was identical to the strain shown as *cup1*Δ + *CUP1his*-OE in **Figure 1C** (*CUP1his* strain). Western blotting analysis showed a clear band with the theoretical molecular weight of Cup1-His only in the *CUP1his* strain, confirming the production of Cup1-His (**Figure 2B**). SDS-PAGE followed by CBB staining showed a thick band with a molecular weight corresponding to Cup1-His in the *CUP1his* strain but not in the WT, suggesting the overproduction of Cup1-His protein. Subsequently, a cell-impermeable derivative of DAF-FM DA, DAF-FM, was incubated with NOC-5 in the presence or absence of yeast cell-free lysate and the fluorescence intensity was monitored over time (**Figure 2C and 2D**). The fluorescence intensity increased slightly more slowly in the presence of lysate from the WT strain than in the presence of the sample containing buffer instead of lysate, but the difference was not statistically significant. Interestingly, the cell-free lysate from the *CUP1his* strain clearly suppressed the time-dependent increase in fluorescence. These results showed that the accumulated Cup1-His in the cell-free lysate from the *CUP1his* strain quenches NO, suggesting that Cup1 functions as an NO scavenger.

### The expression level of *CUP1* does not change under nitrosative stress conditions

**Figure 3:**
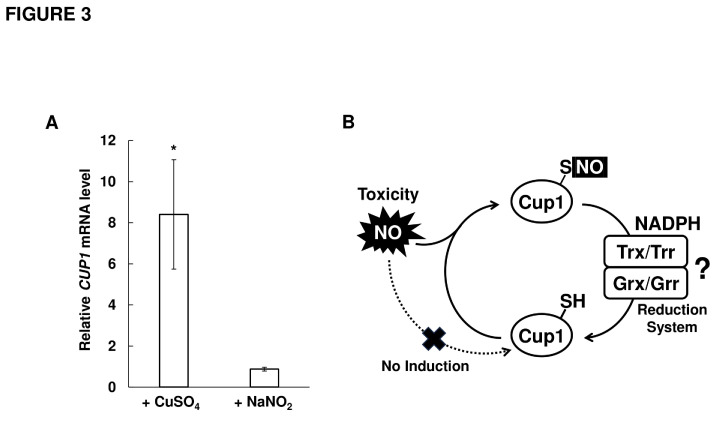
FIGURE 3: Non-inducible nitrosative stress tolerance mechanism mediated by Cup1. **(A)** The *CUP1* mRNA was determined by RT-qPCR in WT strain with or without the exposure to 20 μM CuSO_4_ or 2 mM NaNO_2_ for 1 hour. The relative mRNA level of the *CUP1* gene was calculated with the *CUP1* expression level in the untreated sample as 1.0 using *ACT1* as a reference gene. The values are the means and standard deviations of at least three independent experiments. Statistic significances of differences were analyzed by Student's *t*-test (*p < 0.05, vs the untreated sample). **(B)** The model of nitrosative stress tolerance mechanism by Cup1 in yeast cells. The expression of Cup1 is not induced by NO stimuli. The constitutively or basically produced Cup1 traps NO with its cysteine residue(S) under nitrosative stress conditions. Cup1 modified with NO can be reduced to its unmodified form through Trx/Trr or Grx/Grr reduction system using NADPH as a reducing force.

Genes involved in stress tolerance are often upregulated by the corresponding stress stimuli. Transcription of many genes encoding the proteins involved in NO detoxification, such as fHb and GSNOR, are induced by NO, although NO inhibits the copper- and Ace1-dependent induction of the *CUP1* gene expression [36]. Therefore, we analyzed the transcriptional change of *CUP1* in response to nitrosative stress (**Figure 3A**). We found that the *CUP1* mRNA level was increased 8-fold in response to CuSO_4_ treatment, consistent with a previous study [37]. On the other hand, nitrosative stress induced by acidified nitrite treatment did not change the transcription level of *CUP1*, indicating that *CUP1* expression does not respond to nitrosative stress. These results suggest that the Cup1-dependent NO tolerance mechanism functions as a constitutive NO tolerance system.

## DISCUSSION

A previous report has shown that the overproduction of MT enhanced nitrosative stress tolerance and the MT-encoding gene was upregulated by RNS in mammalian cells [27]. However, it has not been clarified the functions of MT in yeast in relation to nitrosative stress. It has also been unclear whether MT expressed at the basal level or regulated by the endogenous expression system, not only in yeast but also in other organisms including mammalians. In this study, we showed that the yeast MT Cup1 under the physiological expression mechanism contributes to the nitrosative stress tolerance in *S. cerevisiae* cells, by scavenging NO. This is the first report not only to demonstrate the functions of the yeast MT related to NO, but also to indicate that MT with physiological expression regulation contributes to nitrosative stress tolerance.

Cysteine residues in proteins are susceptible to NO-dependent posttranslational modifications, such as *S*-nitrosylation and *S*-glutathionylation. Thus, MT is likely to be *S*-nitrosylated under nitrosative stress conditions, like other proteins with CXXC motifs. A previous study showed that MT was *S*-glutathionylated after nitrosative or oxidative stress treatment in the presence of GSH in mammalian cells [38]. It has also been reported that MT forms intramolecular disulfide bonds under oxidative stress conditions [24], suggesting that Cup1 forms disulfide bonds in it under nitrosative stress conditions, since NO functions as an oxidant. These results suggest that Cup1 is converted into the form with *S*-nitrosylation, *S*-glutathionylation, or an intramolecular disulfide bond in yeast cells in response to nitrosative stress. Whereas, *A. nidulans* nitrosothionein comprises MT-like motif and Trx/Trx reduction system reduces its *S*-nitrosylated cysteine residues to unmodified form. Therefore, some reduction mechanisms, including the Trx/Trr reduction system and/or glutaredoxin/glutaredoxin (Grx/Grr) reduction system, could function coordinately with Cup1 to regenerate the unmodified form of Cup1 by reducing these oxidative modifications (**Figure 3B**).

Our transcriptional analysis demonstrated that the expression level of *CUP1* was not affected by nitrosative stress, which is similar to the finding that GCH2 is not induced by NO [14], and therefore Cup1 would function in a constitutive NO tolerance mechanism. In contrast, the expression of MT is induced by NO stimuli in mammalian cells [27], suggesting that a mammalian MT functions in an inducible defense system against nitrosative stress. The different regulations of MT among host species could be derived from the different amount of MT in the absence of stress stimuli in each organism at least partly. It is possible that the intracellular concentration of Cup1 is sufficient to fully scavenge NO without NO-dependent upregulation in yeast. The difference in the regulation mechanisms of MT can also account for the appropriate response time in yeasts and mammals. The constitutively functioning NO tolerance mechanism involving Cup1 could be necessary and efficient against acute nitrosative stress, which might occur in the living environment of yeast, since this mechanism does not require the time for transcription and translation. On the other hand, the inducible nitrosative stress tolerance mechanism by MT in mammalian cells would be an important factor to attenuate the toxicity of NO generated more slowly, because this inducible system requires the time for transcription and translation for the responsible proteins. Furthermore, the different response of MT to NO stimuli could be related with the particular mechanism by which nitrosative stress exerts its toxicity in the organism. In yeast, the reduced form of Cup1 is decreased by the oxidative modification induced by NO, since the protein level of Cup1 does not increase. Thus, transition metal ions originally bound to Cup1 could be released. However, the amount of transition metal released from MT under nitrosative stress conditions in mammalian cells should be limited more than yeast cells, because the MT protein level is increased in response to NO and thus the protein concentration of unmodified MT is still adequate to trap transition metal ions. Therefore, the disrupted homeostasis of transition metal would be one of the mechanisms involved in NO toxicity. Yeast cells might be more resistant to an increased concentration or disrupted homeostasis of transition metal ion than mammalian cells.

## MATERIALS AND METHODS

### Strains, plasmids, and media

*S. cerevisiae* BY4741 (*MATa his3*Δ*1 leu2*Δ*0 met15*Δ*0 ura3*Δ*0*) [39] was used as WT strain or a host strain to construct yeast mutant strains. BY4741 strain was transformed with a DNA fragment amplified by PCR using the plasmid pFA6a-natNT2 (EUROSCARF) as a template and the primers listed in **Table 1** to construct the *cup1*Δ strain. The DNA fragment encoding Cup1 or Cup1-His was amplified by PCR using a chromosomal DNA of *S. cerevisiae* BY4741 strain as a template with the primers listed in **Table 1** and inserted into the plasmid pAG425GPD-ccdB (Addgene) or pAG426GPD-ccdB (Addgene) by the BP and LR reactions of the Gateway technology (Invitrogen) following the manufacturer's protocol, generating pAG425GPD-CUP1, pAG426GPD-CUP1, pAG425GPD-CUP1-6HIS, or pAG426GPD-CUP1-6HIS. The resulting plasmids pAG425GPD-CUP1 and pAG426GPD-CUP1 were used to overexpress *CUP1* in WT strain. The plasmids pAG425GPD-CUP1-6HIS and pAG426GPD-CUP1-6HIS were introduced into the *cup1*Δ strain to construct the *CUP1his* strain which overproduces Cup1-His. The plasmid pAG425GPD-ccdB and pAG426GPD-ccdB were introduced to WT or the *cup1*Δ strain as empty vectors. In order to compensate the remaining auxotrophic marker, pRS313-MET15 harboring *HIS3* gene from *Candida glabrata* and *MET15* gene from *S. cerevisiae* was introduced each strain [39].

**Table 1.** List of primers used in this study.

**Primer name**	**Primer sequence (5′→3′)**
CUP1-1_Gateway_Fw	GGGGACAAGTTTGTACAAAAAAGCAGGCTTAATGTTCAGCGAATTAATTAACTTC
CUP1-1_Gateway_Rv	GGGGACCACTTTGTACAAGAAAGCTGGGTGTCATTTCCCAGAGCAGC
CUP1-1-6His-Gateway_Rv	GGGGACCACTTTGTACAAGAAAGCTGGGTGTCAGTGATGGTGGTGGTGATGAGATCTAGCTTTCCCAGAGCAGCATG
CUP1_deletion_S1_Fw	GACTGATCTGTTGTACTATCCGCTTCAAATAAATAGATCATTGAAAGGTACGCTGCAGGTCGAC
CUP1_deletion_S2_Rv	GAAAAAAATGTATTACTCAAGACATTCGCTTCTAGGTCAGTCTTCCATCGATGAATTCGAGCTCG
Confrim_deletion_CUP1_Fw	GACTGATCTGTTGTACTATCCGCTTC
Confrim_deletion_CUP1_Rv	CATTCGCTTCTAGGTCAGTCTTC
yACT1 qPCR Fw	CACCAACTGGGACGATATGGA
yACT1 qPCR Rv	GGCAACTCTCAATTCGTTGTAGAA
RT_CUP1-1_Fw	CAATGCCAATGTGGTAGCTG
RT_CUP1-1_Rv	CATTTCCCAGAGCAGCATGAC

Yeast cells were cultured at 25°C in SD medium containing 2% (wt/vol) glucose, 0.5% (wt/vol) ammonium sulfate, 0.17% (wt/vol) Difco^TM^ yeast nitrogen base without amino acids and ammonium sulfate (Becton, Dickinson), with 2% (wt/vol) agar if necessary. pH of SD medium was adjusted to 4.6 or 6.0.

### Quantitative RT-PCR Analysis

Yeast cells cultured until the exponential growth phase in SD medium (pH 4.6) at 25°C were incubated in the absence or presence of 20 μM CuSO_4_ or 2 mM NaNO_2_ for 2 h. Harvested cells were disrupted using Multi-Beads Shocker (Yasui Kikai) with glass beads, and the total RNA was extracted with the RNeasy Mini Kit (QIAGEN) according to the manufacturer's instructions. cDNA was synthesized from the total RNA with the PrimeScript RT reagent Kit (Takara Bio). An RT-qPCR was performed using QuantStudio (Applied Biosystems) and SsoAdvanced Universal SYBR Green Supermix (Bio-Rad Laboratories) with the primer listed in **Table 1**. The *ACT1* gene was used as a reference gene and the relative mRNA level was calculated by the ΔΔCt method. The relative expression level of *CUP1* was calculated with the *CUP1* mRNA level in WT strain or that in the untreated sample as 1.0 in order to compare the *CUP1* expression level among strains or analyze the gene expression in response to stress stimuli, respectively.

### Measurement of the intracellular NO level

Yeast cells grown until the exponential growth phase in SD medium (pH 6.0) at 25°C were treated with 15 μM DAF-FM DA at 25°C for 30 min and then exposed to 1 mM NOC-5 for 1 and 2 h, followed by FCM using BD Accuri C6 Flow Cytometer (Becton Dickinson). To evaluate the intracellular NO level, the relative fluorescence intensity was expressed as a percentage, which were calculated as follows: [(mean of fluorescence intensity at the indicated time point)/(mean of fluorescence intensity at 0 min after NOC-5 treatment)] × 100.

### NO quenching assay

Yeast cells cultured until the log phase in SD medium (pH 6.0) at 25°C were suspended in Lysis buffer containing 20 mM Tris-HCl buffer (pH 7.4), 150 mM NaCl, and 5% (wt/vol) glycerol, followed by cell disruption using Multi-Beads Shocker (Yasui Kikai). Supernatant after centrifugation was used as a cell-free extract. For the crude purification with size exclusion mode, the cell-free extract was subjected to the ultrafiltration using Amicon^®^ Ultra 0.5-ml Centrifugal Filters with 30 K and the flow through fractions were collected. Furthermore, in order to remove small compound in lysate and concentrate protein, the collected fractions were ultrafiltrated with Amicon^®^ Ultra 0.5-ml Centrifugal Filters with 3 K in order to exchange the solvent to Lysis buffer, and the residual fractions were pooled and used as a cell-free lysate in the further analyses. Subsequently, the reaction mixture containing 20 mM Tris-HCl buffer (pH 7.4), 7 μM DAF-FM, 200 μM NOC-5, and the resultant cell-free lysate was incubated at room temperature and the fluorescence was monitored over time by a plate reader TriStar^2^ LB942 (Berthold Technologies) using an F485 excitation filter and an F535 emission filter. The linear slopes from 100 sec to 600 sec after the beginning of reaction was used to calculate the rate of fluorescence increase.

### SDS-PAGE and Western blot analysis

Yeast cells grown until the exponential growth phase in SD medium (pH6.0) at 25°C were suspended in Lysis buffer with 2 mM phenylmethylsulfonyl fluoride and then disrupted by Multi-Beads Shocker (Yasui Kikai). Supernatant after centrifugation with the unified protein concentration was mixed with SDS sample buffer containing 100 mM Tris-HCl (pH 6.8), 20% glycerol, 4% SDS, 0.1% bromophenol blue, and 10% 2-mercaptethanol, and then boiled for 5 min to denature protein. The denatured samples were subjected to SDS-PAGE followed by CBB staining or immunoblotting using Anti-Histidine-Tagged Protein Mouse mAb (13/45/31/2) (Sigma-Aldrich) (anti-His antibody) and Anti-Mouse IgG (H + L), HRP Conjugate (Promega) (anti-mouse antibody). The visualization of immunoblot was performed using Pierce^TM^ ECL Plus Western Blotting Substrate (Thermo Fisher Scientific) and Image Quant LAS-4000 (Fujifilm).

## AUTHOR CONTRIBUTION

Y.Y., R.N. and H.T. conceived the study and designed the experiments. Y.Y. and R.N. performed the experiments. Y.Y., R.N., N.T., and H.T. analyzed the data and wrote the manuscript. All authors read and approved the final manuscript.

## Abbreviations:

MT: – Metallothioein,
NO: – nitric oxide,
RNS: – reactive nitrogen species,
fHb: – flavohemoglobin,
GCH2: – GTP cyclohydrolase II,
GSNO: – S-nitrosoglutathione,
GSNOR: – GSNO reductase,
Trx: – thioredoxin,
Trr: – thioredoxin reductase,
WT: – wild-type,
SD: – synthetic dextrose,
DAF-FM DA: – 4-amino-5-methylamino-2',7'-difluorofluorescein diacetate,
NOC-5: – 3-[2-hydroxy-1-(1-methylethyl)-2-nitrosohydrazino]-1-propanamine,
Grx: – glutaredoxin,
Grr: – glutaredoxin.
